# Neuroendocrine and Cardiovascular Activation During Aggressive Reactivity in Dogs

**DOI:** 10.3389/fvets.2021.683858

**Published:** 2021-08-09

**Authors:** Elena Gobbo, Manja Zupan Šemrov

**Affiliations:** Department of Animal Science, Biotechnical Faculty, University of Ljubljana, Domžale, Slovenia

**Keywords:** dog aggression, physiology, cortisol, serotonin, surface temperature, tail wagging

## Abstract

Our aim was to investigate cardiovascular activation by measuring changes in facial and body surface temperature using infrared thermography, and neuroendocrine activation using salivary cortisol (CORT) and serotonin concentration (SER) in dogs exhibiting aggressive reactivity in real time. Based on two factors, owner-reported past aggressive behaviors, and detailed behavioral observations collected during a Socially Acceptable Behavior test consisting of 16 subtests and, each individual was categorized as aggressive or non-aggressive. CORT and SER showed no difference in neuroendocrine activity between dogs, but aggressive dogs with higher levels of aggression were found to have lower SER. Aggressive dogs also had an increase in facial temperature from pre-test values. The discovery of a correlation between tail wagging and left tail wagging with aggression level and aggression-related behaviors in aggressive dogs is further evidence of the right hemisphere specialization for aggression previously reported in the literature. This study provides the first evidence that both cardiovascular and neuroendocrine systems are activated during an active act of aggression in dogs.

## Introduction

The response of animals to environmental stimuli, often referred to as reactivity (1), varies from individual to individual. When exposed to challenges, animals adopt different individual behavioral strategies or coping styles that are stable over a longer time (2). An individual coping style is an adaptive strategy characterized by a set of behaviors and physiological responses to reduce the impact of a stressor and is characteristic of a particular group of individuals (2). Animals, including dogs, can be described as proactive or reactive copers (3) and exhibit behavioral patterns that can distinguish them as aggressive or non-aggressive individuals, respectively (4). Aggressive reactivity in dogs, especially when directed toward humans, is a widely recognized problem that poses a public health and animal welfare concern (5). The behavior can be classified by its' motivation (territorial-, fear-related etc.) or target (human-, dog-directed etc.), but its' cause cannot always be determined. To date, various physiological factors underlying aggressive behavior have been studied to identify potential biomarkers of aggression, but certain gaps remain. Research (6–9) has primarily focused on comparing groups of dogs with or without a history of owner reported aggressive behavior, and has not aimed to examine physiological activation during an aggressive act. Evidence that considers physiological activation during an actual aggressive reactivity is therefore lacking. For the study of real-time behavior, it is recommended to measure multiple physiological parameters simultaneously (10), non-invasively, so that measurement devices and procedures do not interfere with behavioral responses (11).

Behavioral reactivity to external stimuli has been reported to be associated with cardiovascular parameters such as heart rate (HR), heart rate variability (HRV), and (body and facial) surface temperature. For example, recent evidence suggests that dogs with a history of biting incidents have poorer autonomic regulation, resulting in lower resting HRV (7), while dogs exhibiting aggressive reactivity to threatening stimuli have decreased HRV and increased HR (12). This latter result was based on measurements collected after the stimulus was applied, when the dog was standing still to avoid motion artifacts. In addition to motion artifacts, many researchers agree that the measurement of HR and HRV in moving dogs has other limitations. Sudden bursts of muscle activity during movement can lead to poor electrode conduction (13) and loss of contact or displacement of the electrodes can cause false signals (14). Another limitation is that the monitor strapped around the dog's chest can be intrusive, especially for dogs that are not used to wearing it, so habituation by wearing a dummy monitor may be required (13). An alternative measure that avoids any direct interaction during exercise and potentially alters behavioral responses is infrared thermography, which has been recognized as a useful tool for assessing cardiovascular reactivity in animals, including dogs (15). Findings in animals (16) suggest that it can be used to measure temperature changes associated with positive and negative affective states, as affective states can cause vascular activity that produces changes in heat production and release that lead to changes in surface temperature.

In terms of reactivity during negative affective states, dogs have been shown to have lower nasal temperature while alert when kenneled compared to a home environment (17). Other animal studies showed a decrease in nasal surface temperature in response to threatening stimuli [monkeys: (11, 18)] and a decrease in ocular bulb and periocular area temperature exposed to various stressors [rabbits: (19)]. The only two studies that observed cardiovascular activity during an aggressive act in animals were by Boileau et al. (20) and Rigternik et al. (21) and they reported inconsistent results. Boileau et al. (20) reported a decrease in dorsal surface temperature in pigs during a fight, while Rigternik et al. (21) found no differences between the control group and aggressive dogs that showed human-directed aggression.

The above cardiovascular parameters are closely related to the autonomic stress response “fight or flight”, which prepares an animal to react in a stressful situation (22). Simultaneously, cortisol, the primary stress hormone, is released (23) in conjunction with the production of the inhibitory neurotransmitter serotonin (24). Such neuroendocrine activation modulates cognitive and behavioral functions and determines coping behavior in humans (25) and in non-human animals (26). According to Bari and Robbins (27), serotonin helps both humans and animals to inhibit inappropriate learned behavior and choose adapted behavior. In humans, Montoya et al. (28) found that low serotonin concentration (SER) combined with high testosterone to cortisol concentration ratio (CORT) modulates impulsive aggression. Following owner-reported past aggressive behavior, researchers found that aggressive dogs had significantly lower serum SER levels (6, 8) and higher plasma CORT levels than non-aggressive dogs (9). These studies used an invasive approach when examining cardiovascular activity, which caused unnecessary stress to the animals (29). To avoid this, CORT and SER can be assessed by highly comparable saliva samples (30) and tested during short-term physiological reactivity (31).

Similar physiological reactivity is often reported in the expression of various behaviors. For example, fear and aggression in dogs have different behavioral expression but share similar neurochemistry, resulting in similar physiological reactivity (32). To observe physiological and behavioral parameters in a controlled environment, behavioral tests are the most objective research method. In our study, the Socially Acceptable Behavior (SAB) test, which is known to elicit aggression in aggressively-inclined dogs (33), was used to assess a dog's behavioral phenotype. This test is also known to have a very high predictability of dogs' future biting behavior and a very high correlation between dogs' biting behavior during the test and their biting behavior in the past (33). In our study, we focused on the expression of the behavior and not on motivation for such behavior or target. Police working dogs were selected for the aggressive group because, according to Haverbeke et al. (34), the vast majority of military working dogs behave aggressively during the SAB test. For the non-aggressive group, highly trained dogs (e.g., show, rescue, therapy dogs) known to behave calmly in a new and noisy environment and in the presence of unfamiliar people (35, 36), of the same sex and age were selected. According to the breed nomenclature of the Fédération Cynologique Internationale, the dogs studied were all from the same classified breed group - sheepdogs. A final chosen criterion for inclusion in the dog groups was the behavior shown during the test. We decided to categorize dogs as aggressive if they attacked at least once during the test (37). The dogs primarily placed in the aggressive dogs that failed to exhibit biting behavior and dogs in the non-aggressive group exhibiting biting behavior were excluded from the study.

To provide a comprehensive physiological profile of an aggressive dog, neuroendocrine and cardiovascular parameters were measured simultaneously and non-invasively during the behavioral test of aggressive reactivity. Our main predictions were that aggressive dogs would show neuroendocrine activation measured by increased salivary CORT, but decreased SER, with concomitant cardiovascular activation measured as decreased facial and body surface temperature.

## Materials and Methods

The study was carried out between July and October of 2020 in Ljubljana, Slovenia and was approved by the Administration of the Republic of Slovenia for Food Safety, Veterinary Sector and Plant Protection (U34401-17/2020/10). The dog owners and handlers signed an informed consent form and were given the right to withdraw from the study at any time if the dog showed signs of stress or without giving a reason.

### Animals

Two groups of dogs (aged between 12 and 36 months) with different behavioral backgrounds participated in the study. The aggressive group consisted of 16 male German and Belgian Shepherd police dogs that were reported to have been aggressive during training in the past. The non-aggressive group consisted of 15 male herding dogs of different breeds, trained to behave calmly in new situations and with no known history of aggression by humans. All dogs were without cardiovascular or sensory problems and all, except two in the non-aggressive group, were neutered. The police dogs were recruited through the Slovenian Ministry of Interior, and the privately owned dogs were recruited through Slovenian dog clubs and social media. To reach the test site, 19 dogs that were used to traveling longer distances traveled by car, while others were housed in kennels at the site.

### Behavioral Recordings

**Figure 1:**
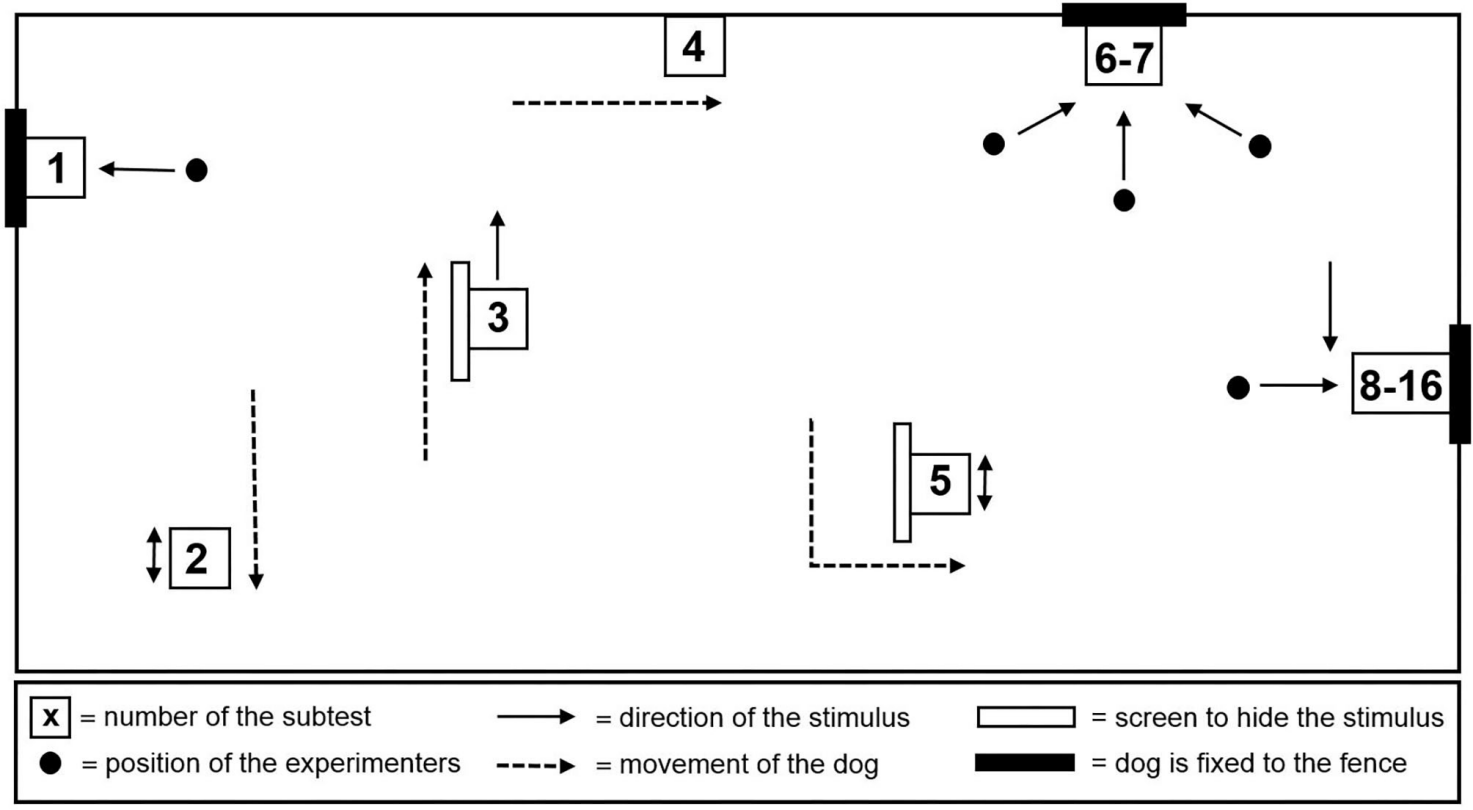
Schematic plan of the test area.

Aggressive reactivity was assessed using the Socially Acceptable Behavior (SAB) test (33). The SAB test consisted of 16 subtests (Supplementary Table 1) and was administered outdoors in a specially set up test area (Figure 1) adapted from Planta and De Meester (33). Each subtest lasted 20 s, with the time in between kept as short as possible. The test was performed by the 3 experimenters. The lead experimenter instructed the owner/handler and guided him through the test, while the other two performed the tasks (e.g., pulling up the blanket). The total duration of the test was approximately 10 min per dog, mainly depending on the dog's cooperation in taking the thermographic images after each subtest. For safety reasons, the dogs were equipped with a harness, a leash, and an additional fixed leash [in subtests (1, 6–16)]. The owner/handler was present during subtests 1 through 7 and 16 and either held the dog on a short leash or the dog was tethered with a double 1.5 m fixed leash.

Behavior was videotaped and coded using the Solomon coder (© 2019 by András Péter). Using the scoring method introduced by van der Borg et al. (37), an aggression and anxiety score was assigned to each dog. During each subtest, aggression was scored on a 3-point scale, with 0 points assigned if there were no signs of aggression, 1 point if the dog showed threat (e.g., growling, baring teeth, staring), and 2 points if the dog attacked (e.g., snapping, biting, lunging). Anxiousness was scored on a 5-point scale indicating whether the behavior was safe (0 points), unsafe (1 point), fearful (2 points), extremely fearful (3 points), and panicky (4 points). Scores were cumulative, with a maximum of 32 points for the aggression score and 64 points for the anxiety score. These scores represented the highest aggression and anxiety scores. More detailed behavioral reactivity during the SAB test was analyzed either as duration or frequency of occurrence using a predefined ethogram (Table 1).

**Table 1:** Descriptive ethogram of the observed behaviors during SAB test.

**Category**	**Behavior**	**Description**	**Scoring**
Locomotion (37)	Moving	Moving with at least one step with each paw	Duration
	Standing	Standing upright, with all four paws on the ground (may move a maximum of two steps)	Duration
	Sitting	Behind is on the ground, forelegs are stretched and support the front of the body	Duration
	Lying down	All four legs and belly are in contact with the ground	Duration
Posture (34, 37)	High	Elevation of the head and/or pointed ears, tail position higher than neutral	Duration
	Neutral	As shown by dogs in neutral conditions, natural position of the tail	Duration
	Low	Bent legs, ears positioned backwards, tail position lower than neutral	Duration
Aggression (34, 37)	Staring	Gazing at the stimulus right in the eyes and freezing of the body	Frequency
	Baring teeth	Showing of the teeth by lifting the upper and lower lips, may be accompanied by nose wrinkling	Frequency
	Snapping	Fast biting movement toward the stimulus, quick head movement (may be accompanied by showing of the teeth, growling, barking), but no physical contact	Frequency
	Attacking	Fast, maximal movement toward the stimulus, biting movement with open mouth or actual bite (may be impossible due to the safety design), may be accompanied by showing of the teeth, growling, barking	Frequency
	Short bark	One single short barking sound	Frequency
	Rapid barking	Loud, repetitive barking sounds (3–4 barks per second)	Duration
	Growling	Low buzzing sound	Duration
	Growl-bark	Barking sounds preceded by growling	Duration
Fear/stress (37, 38)	Fleeing	Accelerated movement toward the opposite direction of the stimulus (more than 1 meter)	Frequency
	Retreating	Movement toward the opposite direction of the stimulus (up to 1 meter)	Frequency
	Stretching leash	Leash is stretched to the maximal length on the opposite direction of the stimulus	Frequency
	Snout licking	Tongue out and moving along upper lip or nose	Frequency
	Yawning	Widely opening of the mouth and inhalation	Frequency
	Whining	High pitched cyclic sounds	Frequency
Interaction with human (39)	Support seeking	Approaching or pushing toward the owner or the handler	Frequency
	Cover seeking	Hiding behind the owner, the handler, orsomething else with respect to the stimulus	Frequency
	Owner/handler seeking	Continuous gaze toward the direction of the owner or handler during subtest 8–15	Duration
Other behaviors (37)	Exploration	Nose positioned within 3 cm of any feature of the physical environment (testing stimuli excluded)	Duration
	Tail wagging	Movements of the tail, from central position to either side	Duration
		Left wag	Frequency
		Right wag	Frequency
	Play behavior	Human-directed play activities such as play bow or tug-of-war	Duration

Reliability coding was performed for 20% of the videos. The consistency between two coders for frequencies using Cohen's Kappa (κ) was 0.87 and for continuous variables using an intra-class correlation coefficient (ICC) was 0.96.

### Sampling and Data Collection of the Salivary CORT and SER

Saliva samples for the assessment of salivary CORT and SER were collected on three occasions. Samples in the home environment (home samples) were collected when the dog was relaxed and resting. Pre-test samples were collected immediately before the start of the SAB test (approximately 5 minutes after arrival at the test area), while post-test samples for SER were collected immediately after the behavioral test and for CORT were collected 20 minutes later, as the dog's CORT peaks approximately 20 minutes after contact with a stressor (40). Samples were collected using commercially available cotton swabs in plastic tubes (Salivette®, Sarstedt, Germany), following the procedure described by Glenk et al. (41). For safety reasons, saliva samples were collected by the owner/handler. To avoid contamination of the samples, the dogs were not allowed to eat or drink for 30 min before sampling and the person collecting the sample wore latex gloves. Cotton swabs were rotated in both sides of the dog's cheek pouch until saturated with saliva, for at least 30 s. The cotton swabs were used to collect samples. Swabs were examined for visible contamination before being placed in plastic tubes and temporarily stored in a freezer at −20 °C before final storage. The samples were stored for 2–3 weeks. To obtain clear saliva, swabs were thawed and centrifuged at 1,500 g for 15 min at room temperature.

Commercial enzyme immunoassay kits (Cortisol free in Saliva ELISA DES6611; Demeditec Diagnostic Gmbh Germany and Serotonin Research ELISA DEE5900; Demeditec Diagnostic Gmbh Germany) were used for the determination of CORT and SER. ELISA kits have previously been used for CORT (42) and SER (43) assessment in dogs. Samples were tested in duplicate (1:10 dilution for SER samples). The sensitivity of the assay was 0.02 ng/mL for CORT and 0.005 ng/mL for SER. Although owners/handlers were familiar with the procedure, they were not always successful in collecting samples and from the total of 144 samples, saliva could not be extracted from 32 samples (22.2, 8.3% in the non-aggressive group and 13.9% in the aggressive group) due to limited sample volume. One outlier (home CORT in the aggressive group) exceeded the mean by more than 13 standard deviations and was removed from the statistical analysis.

### Thermal Imaging Procedure

Surface temperature was measured three times using infrared thermography. Thermographic infrared images were taken with a portable thermographic camera (Optris PI 640). Images of the dog's facial area and body side were taken immediately before entering the test area (pre-test images) and immediately after the test was completed (post-test images). Body image were taken laterally, from a distance of approximately 2 m (from 1.8–2.5 m, depending on the dog's cooperation). The owner/handler stood sideways to the dog (out of the image) and held the dog by the leash. The side of the body from which the picture was taken was balanced between the dogs. Facial images were taken frontally, from a distance of 30–50 cm. Thermal images of the facial area (during the test images) were also taken during SAB the test, after completion of each subtest. As it is known that images taken in the field can be disturbed by the dog's coat characteristics, the distance between the subject and the camera, and environmental factors such as wind and humidity (44–46). Air temperature (°C), humidity (%) and wind (km/h) were measured before taking pre-test images of an individual dog. Dog characteristics (body weight, coat length, and coat color) were also recorded. Thermal images were analyzed using Optris PI Connect software (Rel. 2.15.2219.0). The facial temperature (before, after and during the test) was calculated from the mean values of the warmest points in the image, while the mean value of the observed body side represented the body temperature (before and after the test).

### Statistical Analysis

Statistical analysis was performed using SAS/STAT software, version 9.4 of the SAS System for Windows (© 2002-2012 SAS Institute Inc). After participating in the SAB test, 2 dogs from the non-aggressive group were excluded from the analysis because they showed biting behavior, and there were 5 dogs from the aggressive group that did not show even a single attack. The final non-aggressive group included 13 dogs, while the aggressive group included 11 dogs. Normal distribution for the quantitative traits was determined using the Shapiro-Wilk test. All reported *P*-values that were <0.05 were considered statistically significant or tended to be significant if *P*-values were <0.10. Means with standard deviations (SD) or percentages were calculated for descriptive statistics.

For behavioral analysis, due to low occurrence, the variables teeth baring (*n* = 0 occurrences) and yawning (*n* = 3 occurrences) were excluded, and the variables snapping and attacking, fleeing and retreating, growling, and snarling were combined into snapping/attacking, fleeing/retreating, and barking/ growling. For each variable, the sum of the occurrences collected during the subtests was used. The difference between groups in demographic variables, behavioral variables, and aggression and anxiety scores was examined. Independent samples *t*-test with the instruction PROC TTEST was used to compare normally distributed variables. Mann-Whitney U-test with the statement PROC NPAR1WAY was applied for variables that were not normally distributed.

Temperature change relative to baseline values was used for data analyzes. There were 18 (4.7%) missing values from images during the test due to technical problems. Physiological data were analyzed using the general linear model (GLM) procedure. For the three cardiovascular models (facial temperature, body temperature, and facial temperature during the test), the fixed effect of group (*n* = 2, aggressive and non-aggressive groups), coat color (*n* = 4; brown, black, tricolor, black-brown), side of measurement (*n* = 2; left and right; for the body temperature model only), and subtest (*n* = 16; for the facial temperature during the test model only) on surface temperature was tested. Models also included age, body weight, coat length, humidity, wind, and aggression and anxiety scores as covariates and dog as a repeated measure (for the facial temperature during test model only). For the neuroendocrine models, the fixed effect of group, time of sampling (*n* = 3; home, pre-test, post-test), and age, body weight, aggression score, and anxiety score as covariates were tested at CORT and SER. The dog was included as a repeated measure. When a significant effect was found, the LSMEANS and ESTIMATE statements were used to compare means and estimate contrast between factor levels. To find significant differences when more than two means were compared, a multiple post-*hoc* test Tukey-Kramer was used. Non-significant variables were eliminated and the final model consisted of significant effects only. The final models achieved R-squared values ranging from 0.23 to 0.27.

To test the association between behavioral and physiological variables within dog groups, Spearman's rank correlation was applied using the PROC CORR statement. For this analysis, in addition to behavioral and surface temperature variables, home values (home CORT and SER) and changes between pre- and post-test CORT and SER were used (CORT and SER change). Only strong correlations (r ≥ 0.7) are presented in this manuscript.

## Results

The selected dogs were of similar age (aggressive dogs: *n* = 11 dogs; mean age: 20 ± 4.9 months; non-aggressive dogs: *n* = 13; mean age: 24 ± 7 months, *t* = 1.59, *p* = 0.13) and coat length (4.3 cm ± 0.9 vs. 6.4 cm ± 4; *t* = 1.70, *p* = 0.1), but aggressive dogs were heavier than non-aggressive dogs (33.6 kg ± 3.1 vs. 25.3 ± 9.3 kg; *t* = −2.84, *p* = 0.01). After data inspection, the two neutered dogs within the non-aggressive group did not stand out in their values for all physiological and behavioral parameters compared to the rest.

### Behavioral Testing

Aggressive dogs had a higher aggression score (10.18 ± 6.31) than non-aggressive dogs (0.46 ± 0.66, Z = −4.20, *p* < 0.0001), but an indifferent anxiety score (aggressive dogs = 6.18 ± 2.71; non-aggressive dogs = 8.15 ± 4.81, Z = −0.84, *p* = 0.40). Individual aggressive dogs that snapped or attacked 3 to 26 times per test (mean: 14 ± 6.8) were more likely to show lower body posture, more movement, staring, snapping/attacking, rapid barking, tail wagging, and left tail wagging than non-aggressive dogs (Table 2).

**Table 2:** Behavioral differences in the SAB test between dog groups.

**Category**	**Behavior**	**Group**	**Mean ± SD**	**Value**	***p*-value**
Locomotion	Moving	12	112.08 ± 20.21 161.93 ± 29.79	−4.86^t^	**<0.0001**
	Standing	12	148.68 ± 38.46 125.75 ± 22.45	1.74^t^	0.10
	Sitting	12	34.08 ± 26.92 20.36 ± 16.31	1.47 ^t^	0.16
	Lying down	12	26.35 ± 48.89 11.48 ± 19.63	−0.62^Z^	0.54
Posture	High	12	71.19 ± 64.97 47.77 ± 64.10	0.89^t^	0.39
	Neutral	12	198.40 ± 50.99 178.45 ± 67.63	0.82^t^	0.42
	Low	12	49.86 ± 40.89 87.23 ± 41.99	−2.20^t^	**0.04**
Aggression	Staring	12	0.00 ± 0.00 7.27 ± 4.77	−4.44^Z^	**<0.0001**
	Short bark	12	1.92 ± 3.28 1.46 ± 1.63	−0.82^Z^	0.41
	Rapid barking	12	4.34 ± 11.16 35.92 ± 37.99	−3.26^Z^	**0.001**
	Growling/barking	12	0.85 ± 1.63 0.93 ± 1.41	−0.40^Z^	0.69
Fear/stress	Fleeing/retreating	12	5.31 ± 5.23 2.18 ± 1.40	−1.34^Z^	0.18
	Stretching leash	12	3.23 ± 3.88 1.09 ± 1.64	−1.28^Z^	0.20
	Snout licking	12	15.00 ± 15.20 20.55 ± 13.90	−0.95^t^	0.37
	Whining	12	5.69 ± 10.50 7.55 ± 6.95	−0.83^t^	0.62
Interaction with human	Support seeking	12	1.84 ± 1.99 1.91 ± 1.51	−0.44^Z^	0.66
	Cover seeking	12	0.31 ± 0.63 0.64 ± 0.67	−1.42^Z^	0.16
	Owner/handler seeking	12	11.75 ± 17.24 2.46 ± 6.05	−1.65^Z^	0.10
Other behaviors	Play	12	12.54 ± 21.66 3.40 ± 5.10	−0.67^Z^	0.53
	Exploration	12	23.32 ± 20.32 10.87 ± 6.05	−1.28^Z^	0.21
	Tail wagging	12	53.37 ± 39.08 150.05 ± 53.4	−5.11^t^	**<0.0001**
	Left tail wagging	12	1.39 ± 7.26 13.18 ± 6.88	−4.06^t^	**0.001**

*Group 1 = non-aggressive dogs; group 2 = aggressive dogs*;

t *t-value (t-test)*;

Z *Z-value (Mann-Whitney U test). Bolded values show significant associations*.

### Measurement of the Salivary CORT and SER

Dog groups did not differ in CORT or SER, but timing of sampling influenced CORT (Table 3), with CORT tending to be lower at home than before the test (*p* = 0.06). As shown in Table 3, the covariate anxiety score was significant for CORT and SER, while the aggression score was significant for SER. Dogs with higher anxiety levels had higher CORT and SER, but those with higher aggression levels had lower SER.

**Table 3:** Effects of variables tested on CORT and SER.

	**CORT**			**SER**		
**Continuous variable**	**Estimate**	**F**	***p***	**Estimate**	**F**	***p***
Age	0.03	0.56	0.46	0.03	0.56	0.46
Weight	−0.05	2.73	0.10	0.09	0.09	0.77
Aggression score	0.01	0.05	0.82	−1.00	−3.13	**0.003**
Anxiety score	0.12	2.37	**0.02**	1.29	2.62	**0.01**
**Level variable**	**LSMEANS (ng/mL)**	**F**	**p**	**LSMEANS (ng/mL)**	**F**	**p**
GroupAggressive dogs	2.62	0.10	0.76	24.39	0.10	0.90
Non-aggressive dogs	2.83			25.24		
TimeHome	2.08^a^	2.54	**0.09**	27.05	0.32	0.73
Pre-test	3.29^b^			23.94		
Post-test	2.78^ab^			23.47		

a, b *Values with different superscripts differ significantly*.

*Bolded values show significant associations*.

### Surface Temperature Measurements

Aggressive dogs (Δ = 1.81 °C, LSMEANS = 4.19) had a significantly greater change in facial temperature during the test than non-aggressive dogs (Δ = 0.98 °C, LSMEANS = −0.03, F = 57.75, *p* < 0.0001), but similar facial changes (non-aggressive dogs: LSMEANS = 1.52, aggressive dogs: LSMEANS = 0.03, F = 0.30, *p* = 0.59) and body surface temperature (non-aggressive dogs: LSMEANS = 0.71, aggressive dogs: LSMEANS = 1.19, F = 0.01, *p* = 0.94). The change in facial surface temperature during the test was influenced by three variables (Table 4). Longer coat, lower humidity, and stronger wind increased or tended to increase temperature. Although one effect of the subtest showed a trend, there were no significant changes between subtests (Supplementary Table 2).

**Table 4:** Effects of continuous variables tested on changes (Δ) in surface temperature.

**Variable**	**Δ** **body temperature**			**Δ** **facial temperature**			**Δ** **facial temperature during testing**		
	**Estimate**	**F**	***p***	**Estimate**	**F**	***p***	**Estimate**	**F**	***p***
Age	−0.28	2.04	0.18	−0.06	0.49	0.50	−0.01	0.07	0.79
Weight	−0.03	0.70	0.84	0.08	1.27	0.28	0.00	0.07	0.79
Coat length	−0.08	0.04	0.84	0.14	0.55	0.47	0.11	8.37	**0.004**
Humidity	0.16	2.12	0.20	0.07	2.23	0.16	−0.09	106.70	**<0.0001**
Wind	0.28	0.51	0.49	0.08	0.23	0.64	0.06	3.34	**0.07**
Aggression score	−0.22	0.67	0.40	0.02	0.04	0.85	0.00	0.00	0.98
Anxiety score	0.06	0.05	0.83	−0.17	2.13	0.17	−0.07	0.22	0.64

*Bolded values show significant associations*.

### Relationship Between Behavioral and Physiological Measures

**Figure 2:**
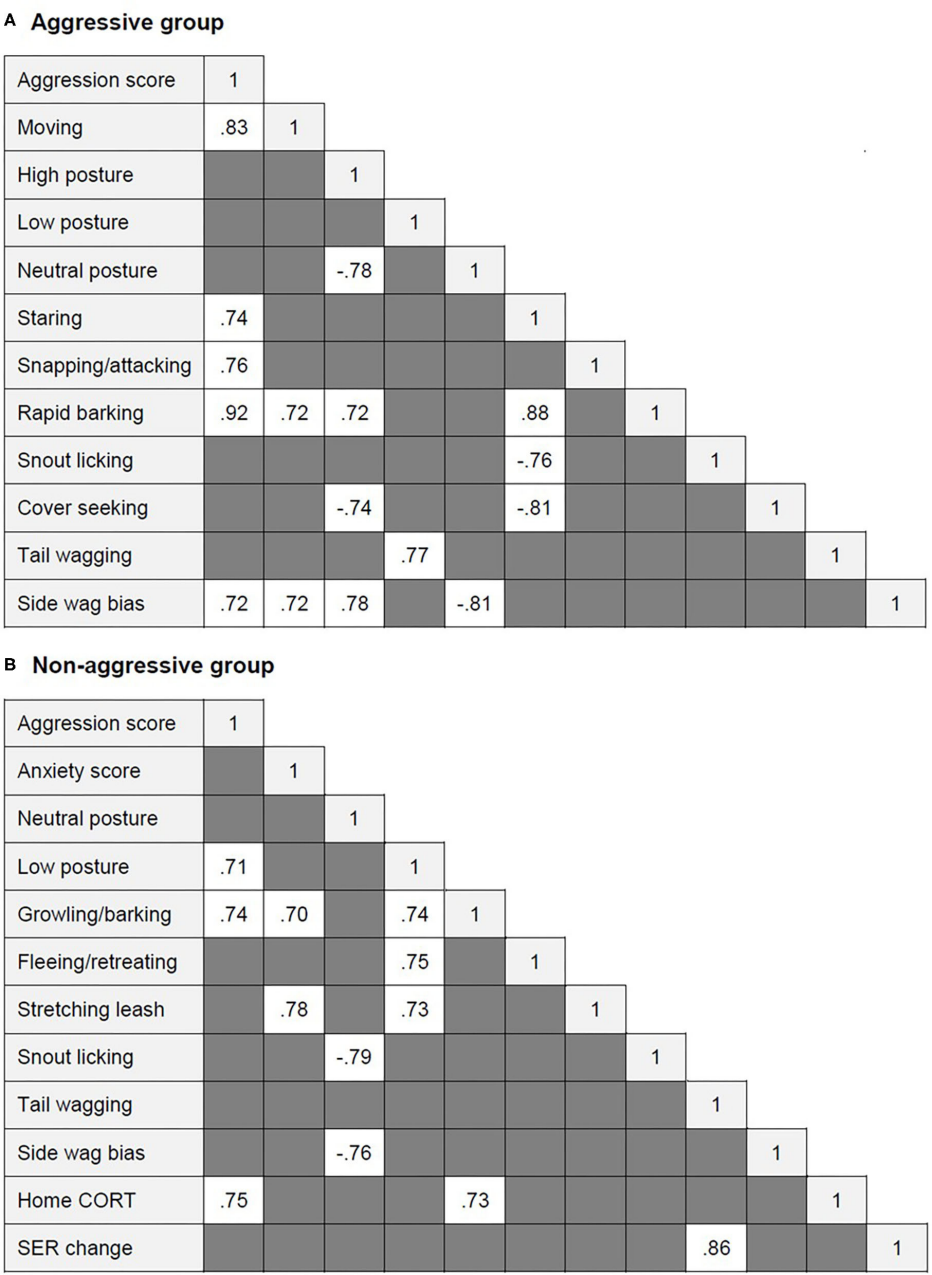
Significant correlations between aggression and anxiety score, behavioral and physiological variables (when r ≥ 0.7) within **(A)** the aggressive group and **(B)** the aggressive group of dogs.

Several highly significant correlations were found within each dog group (Figure 2). In the aggressive group, aggression score correlated positively with moving, staring, snapping/attacking, rapid barking, and left tail wagging. Rapid barking correlated positively with moving, staring, and high posture. Staring correlated positively with rapid barking and negatively with snout licking and cover seeking. Tail wagging was positively correlated with low body posture. Left tail wag (side wag bias) was positively related to aggression score, moving, and high posture and negatively related to neutral posture. High posture was also negatively correlated with neutral posture and cover seeking.

In the non-aggressive group, aggression score was positively correlated with low posture, growling/barking, and higher home CORT. Home CORT also correlated positively with growling/barking. Anxiety score correlated positively with stretching the leash. Low posture correlated positively with growling/barking, fleeing/retreating, and leash stretching. Neutral posture correlated negatively with snout licking and left tail wagging. SER change correlated positively with tail wagging.

Combining behavioral, neuroendocrine, and cardiovascular data, the aggressive dogs in our study were characterized by a lower posture with ears held back. They stared, snapped, or attacked, barked rapidly, and wagged their tails frequently, especially on the left side. The physiological profile included increased facial surface temperature and lower SER.

## Discussion

In this study, simultaneous and non-invasive observation of behavioral, cardiovascular, and neuroendocrine changes during aggressive reactivity provided a profile of an aggressive dog that has never been presented before. Although we cannot fully support our hypothesis of neuroendocrine activation, as we found no difference in the serotonergic system and hypothalamic-pituitary-adrenal (HPA) axis in aggressive dogs compared to non-aggressive dogs, our results suggest that higher levels of aggression are associated with lower levels of salivary SER. We confirmed cardiovascular activation, but the finding of increased facial temperature in aggressive dogs is contrary to our expectations. Our results also confirmed the importance of measuring tail wagging and side wagging in dogs when faced with emotional challenges.

Based on owner-reported history of aggression and display of biting behavior in a standardized dog behavior test, our dogs were successfully divided into the aggressive and non-aggressive groups. The phenotypic description of aggressive dogs with lower posture, ears held back, increased staring, snapping, attacking, and rapid barking was consistent with previous observations (34, 47). In addition to these known behaviors, we also observed and described for the first time an increased frequency of tail wagging and more frequent wagging to the left during aggression. Tail wagging is mainly reported in association with positive affective states in dogs (48, 49). However, Quaranta et al. (50) argued that dogs show asymmetric tail wagging in response to stimuli with different emotional valence. This asymmetry is due to differential activation of left and right brain structures (51). A higher amplitude of tail wagging on the right side has been found for stimuli that dogs perceive as positive, while the left side is perceived as negative (50). Measuring the frequency of tail wagging on each side and finding a high correlation of the amplitude of tail wagging to the left side with aggression level and aggression-related behaviors may indicate that left tail wagging is associated with aggressive behaviors. Because left tail wagging results from right hemisphere activation (51), our findings are consistent with other canine studies (52) and several other animal studies (53) that indicate right hemisphere specialization for the expression of intense emotions, including hostility and aggression. Further behavioral observations show that dogs had similarly low levels of anxiety and indifferent anxiety-related behavior whether or not they were characterized as aggressive. This, coupled with the fact that anxiety and aggression share similar physiological reactivity (32), leads us to believe that the physiological changes observed during the test are related only to aggression-related behaviors. We found that some of these behaviors, particularly aggressive threatening behaviors (e.g., growling, barking), were associated with CORT collected in the home neutral environment, but surprisingly only in dogs that do not normally respond aggressively (i.e., non-aggressive dogs).

To find an increase in pre-test CORT values compared to baseline values prior to test participation, albeit with a weak trend, could indicate emotional arousal rather than emotional valence according to Lewandowski et al. (54). Based on this and the evidence that physiology can be altered simply due to arriving in a new situation and meeting new people (55) or anticipation of an activity (56), we consider it less likely that the pretest release of CORT was triggered by transport-induced stress. Assuming that SER responds rapidly to environmental stimuli (24) and that no changes were found between home and pre-test SER, this further suggests that the factors that altered pre-test CORT did not represent a stressful experience for the dogs.

During testing, our results found similar activation of the stress and serotonergic systems in the two groups of dogs, which is not what would be expected based on the nature of the stimuli presented in the behavioral test (57) and based on previous research. We expected aggressive dogs to show higher HPA axis activity based on documentation in humans (58) or in dogs with a history of aggression (9) and during displays of aggression between dogs (59). Next, we expected these dogs to also have a lower SER because a reduced SER produces a generalized state of hyperirritability and lowers the threshold at which humans and animals respond to provocative stimuli (60). Our results were distinctive due to methodological differences and difficult to compare with other studies. Our dogs were tested during real-time aggression, whereas previous studies compared SER and CORT in dogs with or without owner-reported aggression history. Due to the fact that aggressive dogs are under the influence of an emotional attachment to their owner/handler (61), the owner/handler could represent a stress buffer for our dogs, influencing the dog's behavior and physiology, as previously observed for stimuli with a threatening approach (12), potentially masking the physiological changes that resulted from the aggression.

Because only a single bite attempt during the test was sufficient to classify the dog as aggressive, some dogs exhibited biting behavior on infrequent occasions, while some others attempted to bite up to 26 times during the test. Thus, the variability of aggression within the aggressive group was high. Highly aggressive dogs were found to have a lower SER, which is consistent with studies on dogs with a history of aggression (6, 8, 9). We find this result valid since it is known that SER plays a role in the neural control of aggression as an inhibitory regulator of aggressive reactivity (24) and dogs with a low SER have been associated with impaired impulse control (62). This phenomenon has been described as the serotonin deficiency hypothesis of aggression, demonstrating the inverse relationship between SER and aggression in humans (63) and non-human animals (64). Based on our results, it is reasonable to assume that neuromodulation, expressed as a lower SER, is evident only in dogs that exhibit high levels of aggressive behavior.

In addition to neuroendocrine activation, activation of the sympathetic nervous system leading to lower surface temperature has been documented in several animal studies when animals were presented with various aversive situations [monkeys: (18); dogs: (15, 17); rabbits: (19); pigs: (20)]. To our knowledge, only two studies examined surface temperature in an aggressive context [pigs: (20); dogs: (21)]. When comparing temperature change relative to baseline, Rigterink et al. (21) found an increase in eye temperature in both aggressive and non-aggressive dogs, whereas we found no such changes in facial or body surface temperature, regardless of aggression group. However, we believe that the discrepancy between the results is due to the fact that their aggressive group consisted of only 27% of dogs that showed aggressive reactivity during interaction with an unfamiliar person, whereas in our study all such dogs were included and their temperature changes were observed on a smaller area that is assumed to be highly reactive.

However, we observed an increase in facial surface temperature during an actual act of aggression in aggressive dogs, suggesting that aggression activates cardiovascular activation in real time, but not the stress axis, measured as increased salivary CORT. Assuming that eye temperature increases during both negative stressful experiences (15) and positive experiences in dogs of both sexes (49), the change in surface temperature could reflect emotional arousal but not necessarily emotional valence. This has also been suggested in pigs, where Boileu et al. (20) reported a decrease in dorsal surface temperature in pigs during social aggression in both winning and losing individuals. Ward et al. (65) reported an increase in aggressive behavior in males exposed to exercise-induced arousal, similar to what we found.

Our results further suggest that thermal images taken during the test may be considered a better indicator of cardiovascular activation after an aggressive response than the change in temperature before and after the test. This assumption should be taken with some caution, as an increase in surface temperature could be influenced by physical activity. Our aggressive dogs moved significantly more than non-aggressive dogs and exercise resulting in heat being dissipated through skeletal muscle, could lead to an increase in surface temperature (66). In addition, our study is the first field study of its kind to examine aggressive dogs outdoors, but this can be problematic for optimal thermal imaging data collection. These data are typically conducted indoors or in a controlled environment with constant temperature and humidity (11, 15, 19, 21). Our observation revealed that not only humidity and wind act as potential confounders on surface temperature, but also coat length.

In light of our findings, we believe that future studies of aggressive behavior in dogs should address certain methodological improvements. First, rather than looking for a specific phenotype, 'it would be preferable touse a dog as its own control (67), which could overcome the problems of inter-subject variability. Second, participants in our study found the method of saliva collection challenging, so we believe that an alternative method for easier and safer saliva collection, such as a collection tube or cotton head on a plastic handle (13), should be used, especially if a collecting individual is inexperienced and if the dogs involved are aggressive.

## Conclusions

Although our study faces numerous methodological challenges, it represents an important step in simultaneously investigating animal behavioral and physiological responses in the field and in real time. Our work provides the first evidence that aggressive dogs can be characterized by serotonergic, measured as salivary SER, and cardiovascular features, measured as increased facial temperature, during an actual aggressive act. The discovery of novel aggression-related behaviors such as tail wagging and left tail wagging opens a new avenue for the study of lateralization in the context of aggression.

## Data Availability Statement

The raw data supporting the conclusions of this article will be made available by the authors, without undue reservation.

## Ethics Statement

The animal study was reviewed and approved by Administration of the Republic of Slovenia for Food Safety, Veterinary Sector and Plant Protection. Written informed consent was obtained from the owners for the participation of their animals in this study.

## Author Contributions

EG and MZ: conceptualization, methodology, formal analysis, and writing—review and editing. EG: data collection, data curation, and writing—original draft preparation. MZ: supervision. Both authors approved the submitted version of the manuscript.

## Conflict of Interest

The authors declare that the research was conducted in the absence of any commercial or financial relationships that could be construed as a potential conflict of interest.

## Publisher's Note

All claims expressed in this article are solely those of the authors and do not necessarily represent those of their affiliated organizations, or those of the publisher, the editors and the reviewers. Any product that may be evaluated in this article, or claim that may be made by its manufacturer, is not guaranteed or endorsed by the publisher.
