# Supplementary material for: Neuroendocrine and Cardiovascular Activation During Aggressive Reactivity in Dogs
Source: Front Vet Sci. 2021 Aug 9;8:683858. doi: 10.3389/fvets.2021.683858 (PMC8381274; doi:10.3389/fvets.2021.683858)
Supplement: Supplementary file 1 [file Table_1.DOCX]

Supplementary Material

# Supplementary Figures and Tables

Supplementary Table S1. Descriptions of 16 Socially Acceptable Behavior subtests (33).

| Subtest | Description |
| --- | --- |
| 1 | The dog is approached by one tester and petted with an artificial hand |
| 2 | The dog is exposed to an unfamiliar visual stimuli (a blanked is pulled up and down) |
| 3 | The dog is exposed to an unfamiliar visual stimuli (sudden appearance of a cat on a sledge) |
| 4 | The dog is exposed to an unfamiliar sound (sudden activation of a horn) |
| 5 | The dog is exposed to an unfamiliar sound (sudden rattle of metal cans) |
| 6 | The dog is slowly approached and surrounded by three testers |
| 7 | The dog is rapidly approached and surrounded by three testers |
| 8 | The dog is approached by one tester with a dummy dog |
| 9 | The dog is slowly approached by one tester and petted using an artificial hand |
| 10 | The dog is exposed to an unfamiliar sound (a bell is rang in front of the dog) |
| 11 | The dog is exposed to an unfamiliar visual stimuli (an umbrella is rapidly opened and closed in front of the dog) |
| 12 | The dog is exposed to an unfamiliar visual stimuli (a life-sized doll, standing on top of a sledge is pulled in front of the dog) |
| 13 | The dog is approached by one tester and petted with a doll fixed on a pole |
| 14 | The dog is approached by one tester staring. |
| 15 | The dog is approached by the same tester as in subtest 14 and petted with an artificial hand |
| 16 | The dog is approached by the owner or handler and petted with a doll |

Supplementary Table S2. Effects of variables tested on changes (∆) in surface temperature.

|  | | ∆ body temperature | | | ∆ facial temperature | | | ∆ facial temperature during testing | | |
| --- | --- | --- | --- | --- | --- | --- | --- | --- | --- | --- |
| Variable | Level | LSMEANS | F | p | LSMEANS | F | p | LSMEANS | F | p |
| Coat color | Brown | 1.67 | 3.03 | 0.11 | 0.98 | 0.47 | 0.50 | 2.47 | 1.14 | 0.38 |
|  | Black | 1.38 |  |  | 0.67 |  |  | 1.55 |  |  |
|  | Tricolor | 2.10 |  |  | 0.30 |  |  | 2.21 |  |  |
|  | Black -brown | -2.34 |  |  | 0.21 |  |  | 2.10 |  |  |
| Side | Right | -0.64 | 0.73 | 0.41 | - | - | - | - | - | - |
|  | Left | 2.00 |  |  | - | - | - | - | - | - |
| Subtest | 1 | - | - | - | - | - | - | 1.35 | 1.63 | 0.06 |
|  | 2 | - | - | - | - | - | - | 1.60 |  |  |
|  | 3 | - | - | - | - | - | - | 1.45 |  |  |
|  | 4 | - | - | - | - | - | - | 1.72 |  |  |
|  | 5 | - | - | - | - | - | - | 1.48 |  |  |
|  | 6 | - | - | - | - | - | - | 1.87 |  |  |
|  | 7 | - | - | - | - | - | - | 1.94 |  |  |
|  | 8 | - | - | - | - | - | - | 1.92 |  |  |
|  | 9 | - | - | - | - | - | - | 2.37 |  |  |
|  | 10 | - | - | - | - | - | - | 2.79 |  |  |
|  | 11 | - | - | - | - | - | - | 2.58 |  |  |
|  | 12 | - | - | - | - | - | - | 2.65 |  |  |
|  | 13 | - | - | - | - | - | - | 2.26 |  |  |
|  | 14 | - | - | - | - | - | - | 2.11 |  |  |
|  | 15 | - | - | - | - | - | - | 2.62 |  |  |
|  | 16 | - | - | - | - | - | - | 2.59 |  |  |
